# Neoadjuvant targeted therapy for resectable EGFR-mutant non-small cell lung cancer: Current status and future considerations

**DOI:** 10.3389/fphar.2022.1036334

**Published:** 2022-11-17

**Authors:** Wenliang Liu, Siying Ren, Ying Xiao, Lulu Yang, Chao Zeng, Yan Hu

**Affiliations:** ^1^ Department of Thoracic Surgery, The Second Xiangya Hospital of Central South University, Changsha, China; ^2^ Department of Respiratory and Critical Care Medicine, The Second Xiangya Hospital of Central South University, Changsha, China

**Keywords:** neoadjuvant targeted therapy, EGFR, non-small cell lung cancer, tyrosine kinase inhibitor, CtDNA

## Abstract

Epidermal growth factor receptor (EGFR)-tyrosine kinase inhibitors (TKIs) targeted therapy has become the standard of care for patients with EGFR-mutated metastatic non-small cell lung cancer (NSCLC) on the basis of improved prognosis and reduced toxicities compared with chemotherapy. In view of the therapeutic potential of EGFR-TKIs in EGFR-mutated advanced NSCLC, several scholars have explored the value of preoperative use of EGFR-TKIs in patients with EGFR-mutated resectable NSCLC. However, the field of neoadjuvant targeted therapy for EGFR-mutated resectable NSCLC is currently in its infancy. In this mini-review, we summarize the current evidence on neoadjuvant EGFR-TKIs targeted therapy for resectable EGFR-mutated NSCLC and focus on discussing potential clinical strategies of treating resectable EGFR-mutated patients by preoperative administration of EGFR-TKIs-based multimodality therapy.

## 1 Introduction

Lung cancer remains the leading cause of cancer death in China and worldwide (Sung et al., 2021; Xia et al., 2022). Non-small cell lung cancer (NSCLC) accounts for more than 85% of all lung cancers, of which 30%–40% are resectable (Goldstraw et al., 2016). Radical surgical resection is the cornerstone of standard of care for resectable NSCLC (Duma et al., 2019). However, approximately 25%–70% of patients diagnosed with resectable NSCLC will experience recurrence even after complete resection (Goldstraw et al., 2016).

Neoadjuvant therapy aims to allow the timely treatment of subclinical micrometastatic disease and increase R0 resection rate, ultimately improving prognosis (Hu et al., 2021). However, preoperative administration of chemotherapy resulted in only a 5% improvement in 5-year survival for stage Ib-III NSCLC, compared with surgery alone (Group, 2014). This is equivalent to the survival benefit of adjuvant chemotherapy (Group et al., 2010).

The advent of tyrosine kinase inhibitors (TKIs), including first-generation gefitinib, erlotinib, and icotinib, second-generation afatinib and dacomitinib, and third-generation osimertinib, furmonertinib, and almonertinib, has changed the treatment mode of EGFR-mutant advanced NSCLC. Compared with reversible first-generation TKIs, irreversible second-generation TKIs showed improved progression-free survival (PFS), and in the case of dacomitinib, improved overall survival (OS) at the cost of increased toxicity for EGFR-mutant advanced NSCLC (Park et al., 2016; Mok et al., 2018). Moreover, afatinib had better activity in patients with uncommon EGFR mutations (Ke and Wu, 2016). However, more than 50% of EGFR-mutated NSCLC patients using first or second-generation TKIs developed acquired resistance due to gatekeeper T790M point mutation, leading to the development of third-generation TKIs (Andrews Wright and Goss, 2019). As reported in FLAURA trial, osimertinib can significantly prolong PFS and OS of patients with treatment-naïve EGFR-mutant advanced NSCLC and reduce the risk of central nervous system (CNS) metastasis, compared with first-generation EGFR-TKIs (Ramalingam et al., 2020). Moreover, adjuvant osimertinib showed longer disease-free survival (DFS) compared with placebo in patients with stage Ib-IIIa EGFR-mutant NSCLC (Wu et al., 2020).

The field of neoadjuvant targeted therapy for resectable NSCLC with EGFR mutations is currently in its infancy (Hu et al., 2022). In view of the therapeutic potential of EGFR-TKIs in EGFR-mutated advanced NSCLC, several scholars have explored the value of preoperative use of EGFR-TKIs in patients with resectable NSCLC with EGFR mutations. In this mini-review, we summarize the current evidences on neoadjuvant targeted therapy for resectable EGFR-mutated NSCLC and focus on discussing potential clinical strategies of treating resectable EGFR-mutant patients by preoperative administration of EGFR-TKIs-based multimodality therapy.

## 2 Current status of research on the neoadjuvant targeted therapy with EGFR-TKIs

### 2.1 First-generation EGFR-TKIs

Several single-arm phase II trials have investigated the efficacy and safety of preoperative use of erlotinib or gefitinib in the neoadjuvant setting for patients with resectable NSCLC with EGFR mutations (Zhong et al., 2019; Zhang et al., 2021) (see Table 1). Zhang et al. (2021) included 33 patients with stage II-IIIA EGFR-mutated NSCLC who received neoadjuvant gefitinib therapy and found an objective response rate (ORR) of 54.5% and a major pathological response (MPR) rate of 24.2%, with no serious side effects observed. Xiong et al. (2019) reported the clinical outcomes of 19 patients with stage IIIA-N2 EGFR-mutated NSCLC receiving neoadjuvant therapy with erlotinib, with median PFS and OS of 11.2 and 51.6 months, respectively. Furthermore, ORR, R0 resection rate and pathological downstaging rate was 42.1%, 68.4%, and 21.1%, respectively. The CTONG 1103 study conducted a head-to-head comparison of the efficacy and safety of erlotinib *versus* gemcitabine plus carboplatin as neoadjuvant therapy in stage IIIA-N2 EGFR-mutated NSCLC (Zhong et al., 2019). The results showed that the neoadjuvant erlotinib group was significantly better than the neoadjuvant chemotherapy group in terms of ORR (54.1% vs. 34.3%), MPR rate (9.7% vs. 0) and median PFS (21.5 vs. 11.4 months); however, there was no significant difference in median OS between the two groups. In addition, Chen et al. reported that for patients with stage IIIA EGFR-mutated NSCLC receiving neoadjuvant therapy, erlotinib resulted in a higher ORR (67.4% vs. 44.2%), better pathological response rates (65.1% vs. 41.9%), and lower hematologic toxicities compared to chemotherapy. However, there was no significant difference between the two groups in terms of intraoperative bleeding, time to drainage tube removal, and complication rates (Chen Wenqing et al., 2018). Sun et al. (2020) recently performed a pooled analysis of five published prospective clinical trials assessing the efficacy and safety of neoadjuvant EGFR-TKIs for treatment of EGFR-mutated NSCLC. Although neoadjuvant EGFR-TKIs achieved satisfactory surgical outcomes and favorable toxicity profile, the pathological downstaging and pathological complete response (pCR) rates were quite low.

**TABLE 1:** Neoadjuvant EGFR-TKIs targeted therapy for EGFR-mutated resectable NSCLC with published results.

Study	No.	Stage	TKIs	Drug exposure (days)	ORR (%)	Pathologic downstaging	R0 resection	Pathologic remission	Prognosis (months)	Grade 3-4 AEs
CTONG1103 (Zhong et al., 2019)	37	IIIA	erlotinib	42	54	10.8%	73%	0%	PFS:21.5, OS:45.8	0%
Xiong et al. (Xiong et al., 2019)	19	IIIA	erlotinib	56	42	21.1%	68.4%	0%	DFS:10.3, PFS:11.2, OS:51.6	10.5%
Zhong et al. (Zhong et al., 2015)	12	IIIA	erlotinib	42	58	16.7%	25%	NA	DFS:8.6, PFS:6.9, OS:14.5	16.7%
Rizvi et al. (Rizvi et al., 2011)	21	IA-IIB	gefitinib	21	81	NA	NA	NA	NA	NA
Zhang et al. (Zhang et al., 2021)	35	II-IIIA	gefitinib	42	55	20%	82.8%	pCR: 12.1%	DFS:33.5	0%
Chen et al. (Chen Wenqing et al., 2018)	43	IIIA	erlotinib	63	67	NA	79.1%	NA	OS:56	0%
NCT03433469	27	I-IIIA	osimertinib	28-56	46	NA	NA	MPR:15%, pCR:0	NA	0%
NEOS	40	II-IIIB	osimertinib	42	71	NA	94%	MPR:11%, pCR:4%	NA	7.5%
Leng et al.	17	IA-IIIB	osimertinib	56	88	52.9%	100%	MPR:23.5%, pCR:5.9%	NA	0%
Hu et al. (Hu et al., 2022)	13	IB-IIIB	osimertinib	75	69	1	100%	MPR:0, pCR:0	DFS: 9.5	0%

TKIs, tyrosine kinase inhibitors; ORR, objective response rate; AE, adverse events; NA, not available; PFS, progression-free survival; OS, overall survival; DFS, disease-free survival; MPR, major pathological response; pCR, pathological complete response.

### 2.2 Second-generation EGFR-TKIs

The ASCENT study (NCT01553942) is the only clinical trial reporting the outcomes of the preoperative use of second-generation EGFR-TKIs for treatment of EGFR-mutated locally progressive NSCLC (LV et al., 2018). The study was designed to assess the efficacy and safety of afatinib induction therapy followed by radical chemoradiotherapy or induction chemoradiotherapy followed by surgery decided by sequential MDT evaluation. 19 patients were enrolled in this study, including 10 with potentially resectable stage IIIA and 9 with unresectable stage IIIA/B. All patients received a 2-month course of afatinib induction therapy, and 2 patients were withdrew from this study due to disease progression during afatinib therapy. Imaging evaluation after afatinib treatment suggested an ORR of 58% (11/19), with 7 patients receiving radical chemoradiotherapy and another 10 receiving induction chemoradiotherapy followed by surgery. Postoperative pathology showed MPR in 7 (70%) patients, including pCR in 1 (10%). Although no treatment-related deaths occurred, most patients experienced grade 3/4 toxic reactions, including rash in 6 patients, diarrhea in 5, esophagitis in 3, nausea in 3, pneumonia in 2 and febrile neutropenia in 1. With a median follow-up of 30.6 months, disease recurrence occurred in 3 (30%) and 5 (71.4%) patients receiving surgery and radical chemoradiotherapy, respectively. Median PFS and OS were 34.6 and 69.1 months, respectively, with a 2-year OS of 88%.

### 2.3 Third-generation EGFR-TKIs

#### 2.3.1 Prospective clinical trials

Only two single-arm phase II studies have investigated the feasibility of osimertinib as neoadjuvant treatment for EGFR-mutated resectable NSCLC (Blakely. et al., 2021; Lyu et al., 2022). The NCT03433469 study included a total of 13 patients with EGFR-mutated stage I-IIIA resectable NSCLC who received 1-2 cycles (28 days/cycle) of osimertinib followed by surgery (Blakely. et al., 2021). The results showed 6 (46%) patients achieved partial response (PR) and 7 patients stable disease (SD), with the disease control rate (DCR) of 100%. Postoperative pathology indicated MPR in 2 (15%) patients and no pCR was observed, with rates of pathologic and lymph node downstaging of 69% and 80% (4/5), respectively. Similar to the outcomes of first-generation EGFR-TKIs studies, neoadjuvant osimertinib therapy was well tolerated, with only one patient developing grade 2 treatment-related pneumonia and no serious adverse events observed. Moreover, no unplanned delays in surgery or surgical complications occurred. Interestingly, this study found that most patients who did not respond pathologically to osimertinib carried RNA-binding motif 10 (RBM10) loss-of-function mutations. RBM10, one of the members of the RNA-binding protein family, has been implicated in the carcinogenesis of multiple cancers (Cao et al., 2022). Nanjo et al. further revealed that RBM10 loss-of-function could affect tumor apoptosis by regulating mRNA alternate splicing of the mitochondrial apoptosis regulator Bcl-x, thereby reducing the efficacy of treatment with EGFR-TKIs (Nanjo et al., 2022). In addition, the NEOS study updated its outcomes at ELCC 2022 meeting, reporting that neoadjuvant osimertinib treatment resulted in an ORR of 71.1% and an R0 resection rate of 94% (Lyu et al., 2022). Of the 28 patients evaluated pathologically, 3 (11%) patients achieved MPR, including 1 (4%) patient with pCR, and 13 (46%) patients obtained more than 50% of pathological remission. Moreover, preoperative administration of osimertinib was well-tolerated, with no significant increase in perioperative complications.

#### 2.3.2 Real-world retrospective studies

Leng et al. (Leng et al., 2021) reported the results of a multicenter real-world study (ChiCTR210004995) at 2021 WCLC meeting. They retrospectively analyzed the clinical data of 17 patients with EGFR-mutated stage IA-IIIB NSCLC who received neoadjuvant osimertinib treatment for 1–3 months followed by surgical resection and found an ORR of 88.2%. Among them, 4 (23.5%) patients achieved MPR, including 1 patient achieving pCR (5.9%). Rates of pathological and N2 lymph node downgrading were 52.9% and 42.9%, respectively, with no serious adverse effects or surgical complications observed. Furthermore, we retrospectively analyzed the clinical data of patients receiving neoadjuvant treatment with osimertinib for EGFR-mutated resectable NSCLC at our institute (Hu et al., 2022). Patient demographic characteristics, radiological and pathological response assessment, surgical information and data on complications, toxicities and prognosis were collected. Thirteen patients with a median age of 57 years (interquartile range: 52–64 years) were included in the study, of whom 8 (61.5%) were female. Our study showed ORR, complete resection rate, pathological and lymph node downstaging rate were 69.2% (9/13), 100%, 100% and 66.7% (6/9), respectively. Toxicity data were favorable as well, with no patients experiencing drug withdrawal or surgical delays due to the adverse events or grade 2 or worse adverse reactions. No perioperative deaths was observed and only 3 (23.1%) patients occurred postoperative complications. Moreover, all 12 patients who received follow-up survived without disease recurrence, with a median follow-up time of 9.5 months.

## 3 Future considerations of research on the neoadjuvant targeted therapy with EGFR-TKIs

### 3.1 Is neoadjuvant target therapy with EGFR-TKIs better than neoadjuvant chemotherapy?

We are aware that, based on the promising results of Checkmate 816 trial (Forde et al., 2022), neoadjuvant nivolumab plus chemotherapy has been approved by the FDA for treatment of EGFR/ALK-negative resectable NSCLC and also has been included in the 2022 NCCN V3 edition guideline recommendations. However, there is no conclusive evidence whether neoadjuvant targeted therapy has clinical application value for EGFR-mutated resectable NSCLC and whether neoadjuvant targeted therapy is superior to neoadjuvant chemotherapy. Although the CTONG 1103 study found no significant difference in median OS between the neoadjuvant/adjuvant erlotinib group and the neoadjuvant/adjuvant gemcitabine plus cisplatin group, neoadjuvant/adjuvant erlotinib group outperformed in terms of ORR (54.1% vs. 34.3%), MPR rate (9.7% vs. 0), and median PFS (21.5 vs. 11.4 months). The study was flawed by the small sample size and the relatively longer drug exposure in the neoadjuvant erlotinib group, which affected the credibility of the results (Zhong et al., 2019). Phase 3 RCTs with a higher level of evidence are lacking to confirm the superiority of EGFR-TKIs as neoadjuvant therapy *versus* chemotherapy. Based on relatively higher ORR and lower drug toxicity, in my opinion, neoadjuvant targeted therapy appears to be the better choice compared with chemotherapy (Chen Wenqing et al., 2018). Prospective studies [like NeoADAURA (Tsuboi et al., 2021)] will bring us more information on this topic (see Table 2).

**TABLE 2:** Ongoing clinical trials investigating neoadjuvant EGFR-TKIs in EGFR-mutated NSCLC.

Study	Status	Phase	Stage	No.	Regimen	Primary endpoint
NCT03749213	Recruiting	II	IIIA N2	36	Icotinib 8w + surgery + Icotinib 2y	ORR
NCT04201756	Recruiting	II	III	47	Afatinib 16w + surgery + Afatinib 1y	ORR
NCT05104788	Recruiting	II	II-IIIB N2	27	Icotinib plus Chemo 2 cycles + surgery	MPR
ANSWER (NCT04455594)	Not yet recruiting	II	IIIA N2	168	Almonertinib vs. Erlotinib/chemo	ORR
NeoADAURA (NCT04351555)	Recruiting	III	II-IIIB N2	328	Osimertinib vs. osimertinib plus chemo vs. placebo plus chemo + surgery + investigator choice	MPR
FORSEE (NCT05430802)	Recruiting	II	IIIA-IIIB	40	Furmonertinib plus chemo + surgery	ORR
FRONT (NCT04965831)	Not yet recruiting	II	IIIA-IIIB	40	Furmonertinib 8w + surgery + furmonertinib 3y	ORR
APPROACH (NCT04841811)	Not yet recruiting	II	III	156	Almonertinib 8w + surgery or chemoradiotherapy (by MDT) + almonertinib or not (by ctDNA monitors)	ORR, EFS

ORR, objective response rate; MPR, major pathological response; EFS, event-free survival.

### 3.2 Which is better: Neoadjuvant targeted therapy with third-generation vs. first-generation EGFR-TKIs?

It is well known that osimertinib has showed superior efficacy for treatment of EGFR-mutated advanced NSCLC compared with first-generation EGFR-TKIs, as demonstrated by significantly improving prognosis without increasing toxic side effects and reducing the risk of CNS metastasis (Ramalingam et al., 2020). However, there are no head-to-head prospective studies available to compare third-generation *versus* first-generation EGFR-TKIs as neoadjuvant therapy. The ANSWER study (NCT04455594), prospectively exploring the efficacy and safety of aumolertinib *versus* erlotinib or chemotherapy as neoadjuvant therapy for stage IIIA EGFR-mutated NSCLC is currently recruited and expected to provide more clues to ask this question (Liang et al., 2022).

### 3.3 Can prolonging drug exposure of neoadjuvant targeted therapy improve pCR rate?

Previous studies have shown that first-generation EGFR-TKIs can achieve an ORR of 62%–70% for treatment of EGFR-mutated advanced NSCLC, but radiologic remission usually occurs after 2 months of targeted therapy (Liu et al., 2022). However, several studies such as CTONG 1103 have treated patients with EGFR-mutated resectable NSCLC with a 42-day regimen of neoadjuvant therapy and achieved an ORR of only 54.1%–58.3% (Zhong et al., 2015; Zhong et al., 2019; Zhang et al., 2021). It is possible that short drug exposure time may affect treatment efficacy. Paradoxically, Rizvi et al. used a 21-day neoadjuvant regimen and obtained an ORR of 81% (Rizvi et al., 2011). We believe that this may be related to the fact that the study included only stage IA-IIB patients. An abstract presented at 2022 ASCO annual meeting reported on 4 patients with EGFR mutations and 1 patient with ALK rearrangement who received extended neoadjuvant targeted therapy (treatment continued until radiologic remission stopped) followed by surgical resection (ZH et al., 2022). All five patients achieved pCR with a mean neoadjuvant duration of 185 days (range: 163–248 days); the other four patients with non-driver mutations included in the study had similarly excellent outcomes after extended neoadjuvant chemotherapy, with three patients achieving MPR and another one achieving pCR. Whether prolonged neoadjuvant targeted therapy or even prolonged time until radiological non-remission before surgical intervention can be considered for patients with good response to EGFR-TKIs during neoadjuvant therapy remains to be further investigated.

### 3.4 Does neoadjuvant targeted therapy plus chemotherapy outperform neoadjuvant targeted therapy for treatment of EGFR-mutated resectable NSCLC?

A series of phase II clinical trials reported that neoadjuvant treatment with EGFR-TKIs significantly reduced tumor volume, improved radiologic response, and increased radical surgical resection rates. However, this promising effect of tumor radiological regression did not translate into tumor downstaging or pathologic remission. This result may be highly related to the inhibitory nature of EGFR-TKIs’ anti-tumor mechanism and spatial heterogeneity within the tumor. Feng et al. (2022) recently reported 3 cases of EGFR-mutant stage III unresectable NSCLC who initially received 1-2 cycles of neoadjuvant chemotherapy but were replaced with 2 cycles of neoadjuvant aumolertinib therapy due to poor therapeutic efficacy or intolerance of chemotherapy. Interestingly, two of these patients obtained MPR and another one obtained pCR. The ASCENT study attempted to assess the feasibility of neoadjuvant targeted therapy followed by chemoradiotherapy for potentially resectable EGFR-mutated NSCLC, and found that a 2-year OS reached 88% with a median OS of 69.1 months, 23%–47% of patients experienced dose reduction or discontinuation due to adverse effects (LV et al., 2018). In addition, the high CNS metastasis rate underscored the potential for improved prognosis with third-generation EGFR-TKIs that more easily breach the blood-brain barrier. Previous studies have shown that gefitinib plus chemotherapy significantly improves ORR and prolongs PFS and OS for treatment of EGFR-mutated advanced NSCLC compared with gefitinib alone at the cost of increased toxicities (Hosomi et al., 2020; Noronha et al., 2020). It is worthwhile to consider whether neoadjuvant TKIs plus chemotherapy can be a better treatment option for patients with EGFR mutated resectable NSCLC. The FORSEE study (NCT05430802) is a single-arm phase 2 study aiming to investigate the efficacy and safety of neoadjuvant furmonertinib plus cisplatin and pemetrexed for EGFR-mutated stage IIIA-B resectable NSCLC. Future clinical trials (like FORSEE and NeoADAURA studies) will hopefully reveal the value of neoadjuvant targeted combination chemotherapy with third-generation EGFR-TKIs in EGFR-mutated resectable NSCLC (Tsuboi et al., 2021).

### 3.5 Can circulating tumor DNA dynamic monitoring accurately guide neoadjuvant targeted therapy?

ctDNA monitoring has become a routine non-invasive tool for assessing tumor burden and minimal residual disease (MRD), with value for early diagnosis, efficacy assessment, recurrence monitoring, and prognosis determination. Currently, there is no evidence for ctDNA monitoring to guide neoadjuvant targeted therapy for EGFR-mutated resectable NSCLC. The FRONT study (NCT04965831) has been initiated to explore the efficacy and safety of the neoadjuvant 8-week plus adjuvant 3-year regimen of furmonertinib for treatment of EGFR-mutated stage IIIA-IIIB resectable NSCLC, while tissue and blood ctDNA monitoring will be performed at multiple time points. The APPROACH study (NCT04841811) will prospectively explore the efficacy and safety of induction aumolertinib therapy followed by different adjuvant therapy regimens guided by ctDNA dynamics in the MDT setting for unresectable stage III EGFR-mutated NSCLC. These studies are expected to bring evidence for genetic testing to assess efficacy and guide treatment.

#### 3.6 Do concurrent mutations have impact on the response to neoadjuvant EGFR-TKIs therapy?

Advanced NSCLC studies have shown that concurrent genetic alterations, including common alterations (intra-EGFR co-mutation, TP53, PIK3CA, and PTEN) and driver gene alterations (ALK, KRAS, ROS1, and MET), may affect EGFR-TKI efficacy and partially explain the heterogeneous clinical outcomes (Guo et al., 2020). Little is unknown about the effect of concurrent genetic alterations on the efficacy of neoadjuvant EGFR-TKI therapy. The NCT03433469 study reported RBM10 loss-of-function mutations occurred in three out of four (75%) patients with no evidence of pathological response to neoadjuvant osimertinib therapy. Xiong et al. investigated the effects of concomitant TP53 mutation on prognosis of stage IIIa-N2 patients who received neoadjuvant erlotinib therapy. In their study, seven out of eight EGFR-mutant patients who underwent NGS testing had a combined TP53 mutation. To be noticed, two patients who experienced longer PFS (36 and 38 months, respectively) had no TP53 mutations or very low TP53 abundance. In contrast, the patient with the highest TP53 abundance had a limited benefit in PFS (only 8 months). The limited current evidence indicate the negative impact of co-mutations Clearly, the understanding of whether concurrent mutations affect neoadjuvant EGFR-TKI efficacy is limited by the small number of cases and unclear concurrent mutation status in the existing literature, but it warrants further exploration in the future.

#### 3.7 Will neoadjuvant immunotherapy be another choice for the treatment of EGFR-mutated NSCLC?

Immunotherapy show impaired efficacy in most EGFR-mutated NSCLC patients (Gainor et al., 2016), even in those with high PD-L1 expression (Lisberg et al., 2018). The lack of CD8^+^ tumor-infiltrating lymphocytes in the tumor microenvironment, lower PD-L1 expression and overactivated oncogenic pathway may be possible explannations for the lack of efficacy of PD-L1 inhibitors in these populations (Gainor et al., 2016; Lin et al., 2019). Due to the favorable pathologic response and controlled safety profile, neoadjuvant chemoimmunotherapy currently becomes the standard of care for EGFR/ALK-negative resectable NSCLC. However, little is known about the value of neoadjuvant immunotherapy for EGFR-mutated NSCLC. Two previous phase 2 neoadjuvant chemoimmunotherapy trials reported excellent pathological response in EGFR-mutated NSCLC, which was underpowered by the small sample (Provencio et al., 2020; Shu et al., 2020). Zhang et al. (2022) recently performed a multicenter pooled analysis of 40 oncogene-mutant NSCLC treated with induction immunotherapy. Among the 19 patients harboring EGFR mutation, 8 (42.1%) and 2 (10.5%) patients obtained MPR and pCR, respectively, indicating the potential clinical feasibility of neoadjuvant immunotherapy for resectable EGFR-mutated NSCLC. As known, PD-L1 expression is considered as a predictive biomarker for immunotherapy. Zhao et al. (2022) reported a case of EGFR-mutated NSCLC patient with high PD-L1 receiving chemoimmunotherapy experienced quick disease progression with distant metastasis. Further examination of pretreatment tumor microenvironment revealed rare infiltration of CD8^+^ T lymphocytes, which might explain the primary resistance to chemoimmunotherapy. Comprehensive analysis of driver mutation status, PD-L1 expression, together with tumor immune microenvironment may offer a better prediction of treatment efficacy for EGFR-mutated NSCLC patients, even in the case of high PD-L1 expression (Zhao et al., 2022).

## 4 Conclusion

Compared to the hot-spot neoadjuvant immunotherapy studies in resectable NSCLC, the field of neoadjuvant targeted therapy with EGFR-TKIs is relatively less studied. The current literature suggest that neoadjuvant therapy with EGFR-TKIs significantly reduce tumor volume, improve imaging response, and increase radical surgical resection rate, but does not translate into disease downstaging or pathological remission. Whether RBM10 loss-of-function mutations are co-mutations that negatively modulate the efficacy of neoadjuvant EGFR-TKIs remains to be further explored. The results of clinical studies such as ANSWER and NeoADAURA are expected to guide neoadjuvant targeted therapy strategies for EGFR-mutated resectable NSCLC.
